# The immune checkpoint molecule V-set Ig domain-containing 4 is an independent prognostic factor for multiple myeloma

**DOI:** 10.18632/oncotarget.19468

**Published:** 2017-07-22

**Authors:** Jin Roh, Youkyoung Jeon, A-Neum Lee, Sang Min Lee, YeonMee Kim, Chang Ohk Sung, Chan-Jeoung Park, Jung Yong Hong, Dok Hyun Yoon, Cheolwon Suh, Jooryung Huh, Inhak Choi, Chan-Sik Park

**Affiliations:** ^1^ Department of Pathology, University of Ulsan College of Medicine, Asan Medical Center, Seoul, Korea; ^2^ Department of Oncology, University of Ulsan College of Medicine, Asan Medical Center, Seoul, Korea; ^3^ Department of Laboratory Medicine, University of Ulsan College of Medicine, Asan Medical Center, Seoul, Korea; ^4^ Asan Institute for Life Sciences, University of Ulsan College of Medicine, Asan Medical Center, Seoul, Korea; ^5^ Cell Dysfunction Research Center, University of Ulsan College of Medicine, Asan Medical Center, Seoul, Korea; ^6^ Department of Microbiology and Immunology, Advanced Research Center for Multiple Myeloma, Inje University College of Medicine, Busan, Korea; ^7^ Department of Hematology/Oncology, Busan Paik Hospital, Inje University College of Medicine, Busan, Korea; ^8^ Department of Pathology, Haeundae Paik Hospital, Inje University College of Medicine, Busan, Korea

**Keywords:** multiple myeloma, immune checkpoint, VSIG4, immunohistochemistry, prognosis

## Abstract

Multiple myeloma (MM) remains as an incurable disease, despite recent substantial improvements in treatment. Therefore, development of novel biomarkers for risk stratification and new therapeutic targets are imperative. One of the emerging treatments for MM is the immune checkpoint blockades. V-set Ig domain-containing 4 (VSIG4) is a lately studied B7-related immune checkpoint modulator. We assessed the VSIG4 expression in patients with MM and its prognostic impact. We analyzed 81 bone marrow and 66 extramedullary biopsy samples of MM patients using immunohistochemistry. VSIG4 mRNA expression data from the Multiple Myeloma Genomics Portal (MMGP) were analyzed to validate our results. The overall survival (OS) of the high VSIG4 expression group was significantly poorer than that of the low VSIG4 expression group (*p* = 0.046). VSIG4 expression was remained statistically significant after adjustment for revised international staging system (rISS) and Mayo stratification algorithm (mSMART) risk classification, respectively (*p* = 0.019 and 0.017). Corroborating results were also observed on analyses of VSIG4 expression in patients with extramedullary MM and external data from the MMGP. Our results suggest that VSIG4 expression in MM is an independent indicator of poor prognosis, implying a possible therapeutic target for immunotherapy for MM.

## INTRODUCTION

Multiple myeloma (MM) is the second most common hematologic malignancy with clonal expansion of terminally differentiated plasma cells. MM accounts for 10–15% of all hematologic malignancies and 20% of deaths from hematologic malignancies [1]. Although substantial improvements have been made in the treatment of MM, including the use of autologous stem cell transplantation (ASCT), immunomodulatory molecules (IMiDs), and proteasome inhibitors, MM remains as an essentially incurable disease. The heterogeneity of the disease, which comprises 7 or 8 different subgroups, has impeded the establishment of a standard treatment [2–4]. Furthermore, there is a lack of histologic markers to accurately predict prognosis. These statements highlight the need for new target molecules for better risk stratification of the disease and for the development of novel therapeutic modalities.

Immune checkpoints—also known as co-inhibitory molecules, including B7-H1 (program death-ligand 1, PD-L1), B7-H3, B7-H4, programmed death-1 (PD-1), and cytotoxic T-lymphocyte-associated antigen 4 (CTLA-4) —play crucial roles in maintaining self-tolerance and limiting immune-mediated tissue damage under physiologic conditions [5]. The overexpression of these co-inhibitory molecules is associated with disease progression and cancer-specific death in many human cancers, including pancreatic carcinoma [6], non-small cell lung carcinoma [7], renal cell carcinoma [8], squamous cell carcinoma of the head and neck [9], and malignant melanoma [10]. Furthermore, immune checkpoint inhibitors have shown significant antitumor activity in various types of malignancies. Clinical trials of immune checkpoint inhibitors targeting CTLA-4, B7-H1,and PD-1 have indicated promising results for the treatment of many types of cancers including hematologic malignancies [5, 11–16].

In particular, MM has also been studied as a target of immunotherapy [17]. Görgün *et al*. described the anti-MM immune response induced by the PD-1/PD-L1 blockade alone and in combination with lenalidomide [18]. A phase 1 study of anti-PD-1 antibody (CT-011) has reported promising results on MM patients [19] and anotherphase-1 study of PD-1 inhibitor (Pembrolizumab) on MM (NCT01953692) is also currently ongoing [12]. In addition to PD-1 or CTLA-4, other co-inhibitory or co-stimulatory molecules, such as 4-1BB(CD137), lymphocyte-activation gene 3 (LAG-3, CD223), OX40 (CD134), and T-cell immunoglobulin and mucin-domain containing-3 (TIM-3), are being studied as potential targets for antitumor immunotherapy [20].

The V-set Ig domain-containing 4 (VSIG4, also referred to as CRIg or Z39Ig) is a recently studied immune checkpoint molecule which belongs to B7-related family member. VSIG4 is physiologically expressed on tissue-resident macrophages, including alveolar macrophages in the lung and Kupffer cells in the liver. It shares a set of conserved amino acids with the B7 family members, and contains 1 complete IgV-type domain and a truncated IgC-type domain [21, 22]. VSIG4 has been known to block the alternative complement pathway by binding to the convertase subunit C3b [22]. Moreover, it inhibits CD4+ and CD8+ T cell proliferation by ligating an unknown receptor to the T cells [21]. Initially, VSIG4 expression has been studied regarding the pathogenesis of inflammatory diseases such as rheumatoid arthritis, atherosclerosis, and chronic HBV-hepatitis [23, 24]. However, recent studies reported that VSIG4 expression is involved in lung cancer development and associated with poor prognosis of high grade glioma [25, 26]. In the present study, we assessed the VSIG4 expression in patients with MM and evaluated its prognostic impact. We demonstrated that high VSIG4 expression was significantly correlated with poor survival.

## RESULTS

### Patient characteristics

The general characteristics of the patients are summarized in Table 1. The mean patient age at the time of diagnosis was 62.0 years (range, 44–79 years). The number of male and female patients was similar in the study population (41 and 40, respectively). The mean follow-up duration of this cohort was 40.3 months (range, 0–96 months). Sixty-two patients (76.5%) died due to the disease at the time of the study. The OS rates for the MM patients were 76% at 1 year, 61% at 2 years, and 40% at 5 years, with overall median survival duration of 39.2 months. The characteristics of patients whose specimens were obtained via extramedullary biopsies are summarized in Supplementary Table 1.

**Table 1:** Characteristics of multiple myeloma patients

		Total (%)
Age (years)	< 55	17 (21.0)
	≥ 55	64 (79.0)
Sex	male	41 (50.6)
	female	40 (49.4)
Monoclonal Ig	Heavy chain	
	IgG	29 (35.8)
	IgA	21 (25.9)
	IgD	3 (3.7)
	free	28 (34.6)
	Light chain	
	κ	46 (56.8)
	λ	35 (43.2)
Durie-Salmon stage	1	12 (14.8)
	2	12 (14.8)
	3	57 (70.4)
ISS	1	14 (17.3)
	2	32 (39.5)
	3	35 (43.2)
rISS	1	11 (13.5)
	2	60 (74.1)
	3	9 (9.9)
	Undetermined	2 (2.5)
mSMART risk stratification	standard risk	50 (61.7)
	Intermediate risk	25 (30.9)
	High risk	4 (4.9)
	Not performed	2 (2.5)
Chromosomal abnormality	Absent	37 (45.7)
	Present	42 (51.9)
	Not performed	2 (2.5)
Treatment	ASCT	
	Not performed	50 (61.7)
	Performed	31 (38.3)
	Novel agents	
	Not used	41 (50.6)
	Used	40 (49.4)
plasma cells in bone marrow (mean, %) (±SD)		33.32 (±24.33)
24 hour urine protein (mean, mg/day) (±SD)		2336.88 (±3329.75)
serum calcium (mean, mg/ℓ) (±SD)		9.28 (±1.29)
hemoglobin (mean, g/ℓ) (±SD)		10.13 (±2.10)
serum lactate dehydrogenase (mean, IU/L) (±SD)		237.91 (±103.86)
β2-microglobulin (mean, mgZ/ℓ) (±SD)		8.34 (±11.03)
serum albumin (mean, g/ℓ) (±SD)		3.21 (±0.75)

ASCT: Autologous stem cell transplantation, ISS: International Staging System; rISS: revised ISS, mSMART: Mayo Stratification of Myeloma and Risk-Adapted Therapy; SD: standard deviation

### Correlation between VSIG4 expression and clinicopathologic characteristics

The patients were divided into two groups according to their histologic score: the low expression (score: 0–16) group and high expression (score: 17–27) group. Among the 81 patients, 35 (43.2%) were categorized as the high VSIG4 expression group. The correlation between VSIG4 expression and clinicopathologic factors was examined (Table 2). High VSIG4 expression was significantly associated with female sex (*p*= 0.014) and higher rISS (*p*=0.032). Patients with high ISS also had tendency of high VSIG4 expression (*p* = 0.083). However, there was no significant correlation between VSIG4 expression and other clinicopathologic characteristics, such as age, immunoglobulin (Ig) restriction, mSMART risk stratification, treatment, chromosomal abnormality, 24h urine protein level, the percentage of bone marrow plasma cells, serum calcium level, serum hemoglobin (Hb) level, serum lactate dehydrogenase (LDH) level, serum β2-microglobulin level, and serum albumin level.

**Table 2:** VSIG4 expression and associations with clinicopathological factors in multiple myeloma patients

Variables		VSIG4 expression		
		Low (%)	High (%)	*p* value
Age (years)	< 55	7 (8.6)	10 (12.3)	0.14
	≥ 55	39 (48.1)	25 (30.9)	
Sex	male	29 (35.8)	12 (14.8)	0.01
	female	17 (21.0)	23 (28.4)	
Monoclonal Ig	Heavy chain			0.25
	IgG	14 (17.3)	15 (18.5)	
	IgA	14 (17.3)	7 (8.6)	
	IgD	3 (3.7)	0 (0.0)	
	free	15 (18.5)	13 (16.0)	
	Light chain			0.34
	κ	24 (29.6)	22 (27.2)	
	λ	22 (27.2)	13 (16.0)	
Durie-Salmon stage	1	7 (8.6)	5 (6.2)	0.36
	2	9 (11.1)	3 (3.7)	
	3	30 (37.0)	27 (33.3)	
ISS	1	7 (8.6)	7 (8.6)	0.08
	2	23 (28.4)	9 (11.1)	
	3	16 (19.8)	19 (23.5)	
rISS	1	4 (5.1)	7 (8.9)	0.03
	2	39 (49.4)	21 (26.6)	
	3	2 (2.5)	6 (7.6)	
mSMART risk stratification	standard risk	30 (38.0)	20 (25.3)	0.39
	Intermediate risk	14 (17.7)	11 (13.9)	
	High risk	1 (1.3)	3 (3.8)	
Chromosomal abnormality	Absent	24 (30.4)	13 (16.5)	0.18
	Present	21 (26.6)	21 (26.6)	
Treatment	ASCT			0.46
	Not performed	30 (37.0)	20 (24.7)	
	Performed	16 (19.8)	15 (18.5)	
	Novel agents			0.31
	Not used	21 (25.9)	20 (24.7)	
	Used	25 (30.9)	15 (18.5)	
24 hour urine protein (mg/day)		1904.95	2944.28	0.22
(±SD)		(±2480.58)	(±4218.55)	
plasma cells in bone marrow (mean, %)		31.44	35.79	0.43
(±SD)		(±26.62)	(±21.07)	
serum calcium (mg/ℓ)		9.11	9.50	0.19
(±SD)		(±1.14)	(±1.46)	
hemoglobin (g/ℓ)		10.46	9.70	0.10
(±SD)		(±2.20)	(±1.92)	
serum lactate dehydrogenase (IU/L)		242.30	232.14	0.67
(±SD)		(±112.88)	(±91.96)	
β2-microglobulin (mg/ℓ)		7.06	10.02	0.23
(±SD)		(±6.22)	(±15.16)	
serum albumin (g/ℓ)		3.32	3.07	0.13
(±SD)		(±0.65)	(±0.86)	

ASCT: Autologous stem cell transplantation, ISS: International Staging System; rISS: revised ISS, mSMART: Mayo Stratification of Myeloma and Risk-Adapted Therapy; SD: standard deviation

In extramedullary biopsy, older age (≥ 55) and not performing ASCT were significantly associated with high VSIG4 expression (*p* = 0.011 and 0.001, respectively). Other clinical factors including sex, ISS, results of cytogenetics were not associated with VSIG4 expression. These results are summarized in Supplementary Table 1. For 11 of the total patients included in the study, we could analyze VSIG4 expressions in both bone marrow and extramedullary biopsies. There was no statistically significant difference in the VSIG4 expression between bone marrow and extramedullary biopsies in the same patient (*p* = 0.196).

### Patient survival and VSIG4 expression

**Figure 1:**
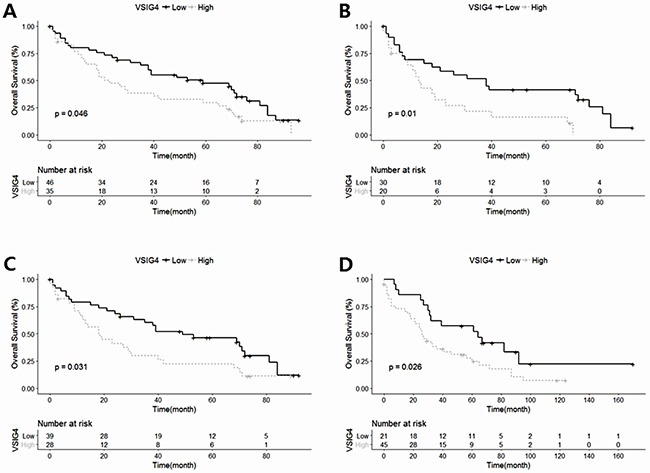
Kaplan–Meier survival curves in each multiple myeloma subgroups classified according to V-set Ig domain-containing 4 (VSIG4) protein expression **(A)** The overall survival rate is charted according to VSIG4 expression in the entire cohort. **(B)** The overall survival rate in autologous stem cell transplantation-ineligible patients according to VSIG4 expression. **(C)** The overall survival rate in International Stage System 2/3 patients according to VSIG4 expression. **(D)** The overall survival rate in patients with extramedullary multiple myeloma.

The OS of the high VSIG4 expression group was significantly poorer than that of the low VSIG4 expression group (*p* = 0.046, log-rank test, Figure 1A). However, the EFS of the high VSIG4 expression was not significantly different with that of the low VSIG4 expression group (*p* = 0.321, log-rank test). The covariates for multivariate analysis were selected according to the results of the univariate analysis, based on the clinical significance. In univariate analysis, not performing ASCT (*p* = 0.004), high mSMART risk (*p* = 0.004), high ISS (*p* = 0.008), high serum LDH level (*p* = 0.004), high urine protein level (*p* = 0.002), and low serum Hb level (*p* = 0.020) were significantly associated with poorer OS. High rISS stage showed tendency of poorer OS (*p* = 0.050). Although rISS shows marginal statistical significance, we included it as a covariate in multivariate analysis considering its clinical significance. However, the urine protein level was excluded as a covariate for multivariate analysis due to no change of hazard ratio (HR = 1.000). Because serum LDH level was included in rISS, serum LDH level was excluded from covariates when rISS was used as a covariate for multivariate analysis. In addition, the ISS was excluded from covariates because it is included in both rISS and mSMART risk classification. VSIG4 expression was remained statistically significant after adjustment for rISS and mSMART risk classification, respectively (*p* = 0.019 and 0.017) (Table 3).

**Table 3:** Multivariable analysis of VSIG4 expression and characteristics in multiple myeloma patients

Variable		Hazard ratio	95% confidence interval		*p* value	Hazard ratio	95% confidence interval		*p* value
			lower	upper			lower	upper	
VSIG4	low	1.00			0.02	1.00			0.02
	high	1.94	1.09	3.45		1.95	1.12	3.40	
ASCT	not performed	1.00			0.04	1.00			0.01
	performed	0.51	0.27	0.97		0.45	0.24	0.83	
rISS	1					1.00			0.43
	2					1.62	0.58	4.47	
	3					2.34	0.65	8.45	
mSMART risk	standard risk	1.00			0.01				
	Intermediate risk	2.35	1.29	4.27					
	High risk	2.93	0.91	9.39					
serum Hb		0.95	0.81	1.10	0.46	0.96	0.83	1.10	0.55
serum LDH		1.00	1.00	1.01	< 0.01				

rISS: Revised International Staging System; ASCT: autologous stem cell transplantation; LDH: lactate dehydrogenase; mSMART: Mayo Stratification of Myeloma and Risk-Adapted Therapy

We further performed a subgroup analysis by stratifying patients by ASCT status and ISS. In the patients subgroup who had not received ASCT, the high VSIG4 expression group showed significantly poorer OS than the low VSIG4 expression group (*p* = 0.010, log-rank test) (Figure 1B). However, the statistical significance of VSIG4 expression was lost in the patients subgroup who had received ASCT (*p* = 0.315). In addition, the high VSIG4 expression cases with ISS 2/3 also showed a significantly poorer OS as compared to the low VSIG4 expression group (*p* = 0.031, log-rank test) (Figure 1C). In ISS1 subgroup, however, the high VSIG4 expression group did not showed difference in OS with the low VSIG4 expression group (*p* = 0.475). Based on these results, we hypothesized that VSIG4 expression may be more influential in advanced stage disease. To evaluate the prognostic impact of VSIG4 expression in the advanced stage of MM, we performed survival analysis with extramedullary MM biopsies. The VSIG4 expression in extramedullary biopsy specimens was analyzed by using the same method and cut-off value. In the cases of extramedullary MM, the high VSIG4 expression patients exhibited significantly worse OS as compared to the low VSIG4 expression patients (*p* = 0.026, log-rank test) (Figure 1D).

**Figure 2:**
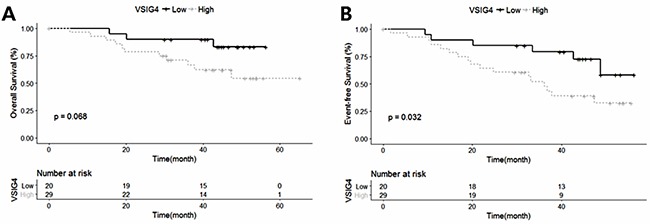
Kaplan–Meier survival curves of the data set obtained from the Multiple Myeloma Genomics Portal database **(A)** The overall survival rate is charted according to VSIG4 expression. **(B)** The event-free survival rate is charted according to VSIG4 expression.

To corroborate our results regarding the association between high VSIG4 expression and poor survival in MM, we analyzed an external data set obtained from the MMGP database. We found that the high VSIG4 mRNA expression group showed significantly poorer EFS as compared to the low expression group (*p* = 0.032) (Figure 2B). Moreover, patients with high VSIG4 mRNA expression tended to have a worse OS than those with low expression (*p* = 0.068) (Figure 2A).

## DISCUSSION

In the present study, we evaluated the prognostic impact of VSIG4 expression in MM using IHC. We found that VSIG4 expression was an independent predictor of a poor prognosis in terms of OS. This prognostic impact became more definite after adjustment for other clinical covariates. These findings are supported by the data obtained from the MMGP database.

VSIG4 is a recently studied immune checkpoint molecule, whose expression is reported to be restricted to resting tissue macrophages including Kupffer cells in the liver [21, 22]. In a previous study, we demonstrated that Kupffer cell-associated VSIG4 plays a crucial role in the maintenance of T and NKT cell tolerance in the liver [27]. In this context, most previous studies on VSIG4 have focused on its expression on macrophages in the tissue microenvironment [25, 27]. Liao *et al*. reported that tumor-associated macrophages (TAM) in lung cancer had upregulated VSIG4 expression, indicating that TAM VSIG4 suppresses tumor-specific T cell functions [22]. However, in our present study, we observed that VSIG4 expression in neoplastic plasma cells in MM was an unfavorable prognostic indicator. In fact, clinical implication of VSIG4 expression by cancer cells has been described recently. Xu *et al*. reported the presence of VSIG4 expression in high-grade glioma cells, which is also associated with a poor prognosis [26]. In parallel, PD-L1 which is expressed on the antigen presenting cell as a ligand of PD-1 is known to be upregulated in many human cancer cells themselves [7, 28, 29]. PD-1 expression in tumor cells, in addition to tumor infiltrating lymphocytes, also has emerged as a new interest [10]. Further studies regarding the role of VSIG4 expression in tumor cells are warranted.

Of note, the impact of VSIG4 expression on survival was vanished in the patients subgroup who had received ASCT (*p* = 0.315), while the high VSIG4 expression group in patients who had not received ASCT had a significantly poorer prognosis (*p* = 0.010) as compared to cases with low VSIG4 expression in the same subgroup. Considering massive alterations in the immune system resulted from ASCT and immune suppression throughout the period of treatment, this data implies the possible contribution of VSIG4 expression in anti-myeloma immunity. Although treatment with newly developed agents such as thalidomide, bortezomib, and lenalidomide can improve OS and delay disease progression in patients who are not eligible for ASCT, a more effective treatment strategy is in demand [30]. VSIG4 can be a plausible candidate target for anti-cancer immunotherapy in addition to stratifying high risk patients by using VSIG4 expression.

In addition to ASCT-ineligible group, the high VSIG4 expression group in patients with high ISS (2 or 3) or extramedullary MM showed significantly poorer OS than the low VSIG4 expression group (*p* = 0.031 and 0.026, respectively). Based on these results, we can postulate that VSIG4 has more prognostic impact in the advanced stage of MM. Although there have been various options for high risk MM including lenalidomide and bortezomib-based regimens, the best treatment strategy has not established yet [31]. Stratifying high risk patients using VSIG4 expression may be a helpful tool for patients with high ISS (2 or 3) or extramedullary MM.

Protein kinase Cα (PKCα) is reported to control VSIG4 expression [32]. PKC signaling pathway is also associated with cell proliferation, survival, and migration of MM [33]. Abnormal PKC signaling pathway may be responsible for VSIG4 overexpression in MM. Cytokines those are involved in PKC signaling pathway such as Tumor necrosis factor α may be responsible for VSIG4 overexpression. In addition, genetic alterations including mutations or translocations of MM may induce constitutive expression of VSIG4. Further studies to explicit the mechanism of VSIG4 induction in MM is necessary.

*Vsig4* is located on the long arm of the X chromosome [34]. Interestingly, significantly more female patients than male patients were in the high VSIG4 expression group (*p* = 0.010). However, OS was not different between female and male patients (*p* = 0.999). These results suggest a possible influence of patient sex in anticancer immunity. Some studies contended that X-linked genes are responsible for the divergence between male and female immune responses [35]. Sex disparity in efficacy of immune checkpoint blockade is also reported [36]. These descriptive data in the current study have limitation to make a conclusion. However, further studies to understand biological differences between males and females in cancer immunology will be needed.

In conclusion, high VSIG4 expression in MM patients is an independent indicator of a poor prognosis. VSIG4 expression, particularly in advanced disease, can help predict and stratify patients with MM. VSIG4 may represent a promising target molecule for novel MM immunotherapies.

## MATERIALS AND METHODS

### Patients and samples

We examined the samples collected from 110 patients diagnosed with MM between 2008 and 2010 at Asan Medical Center. The updated criteria for the diagnosis of MM were applied [37]. Cases without any available initial clinical information were excluded. Cases of localized plasma cell lesions without bone marrow involvement or biopsy specimens obtained at relapse were also excluded. Finally, a total of 81 bone marrow biopsy specimens were included in the analysis. Two expert hematopathologists verified the diagnosis of MM by reviewing the biopsy slides including special studies and clinical information. Clinical information was obtained from the medical records, including sex, age, immunoglobulin light and heavy chain restrictions, treatment details, presence of chromosomal abnormality, serologic markers, and 24h urine protein levels. Chromosomal abnormality was determined according to the results of cytogenetic studies which were composed of karyotype analysis and/or fluorescence in situ hybridization (FISH) analysis for IgH/FGFR3 rearrangement, IgH/CCND1 rearrangement, IgH/MAF rearrangement, 13q deletion, and TP53 deletion. Due to the retrospective nature of this study, the treatments differed among the cases. Of the treatments administered, ASCT and newer agents such as IMiDs (e.g., thalidomide and lenalidomide) and proteasome inhibitors (e.g., bortezomib) were selected as specific covariates. Conventional cytogenetic and/or FISH tests were performed for risk stratification. Durie Salmon (DS) stage, International Stage System (ISS), revised ISS (rISS), and the risk stratification used in the Mayo stratification algorithm (mSMART) [38] were applied for the classification of patients. The medical records were retrospectively reviewed and the clinical features, pathologic findings, cytogenetic results, and clinical outcomes of the patients were evaluated. To estimate the overall survival (OS) and event-free survival (EFS) rate, patients were followed from the date of diagnosis to the date of death. The cause of death was also recorded. To analyze the VSIG4 expression pattern in advanced stage of MM, extramedullary biopsy materials were also collected from 66 MM patients between 2000 and 2011 at the Asan Medical Center. For these patients, clinical information including age, sex, ISS, whether ASCT was performed, result of cytogenetics was also collected. The study protocol was approved by the Institutional Review Board (project number 2015-0751) of the Asan Medical Center.

### Immunohistochemistry (IHC) and quantification of IHC results

Immunohistochemical staining was performed on selected formalin-fixed, paraffin-embedded tissue blocks. Each staining was conducted using auto immunostainer BenchMark XT (Ventana Medical Systems, Tucson, AZ, USA) according to the manufacturer's instructions and by using the reagents supplied with the kit. In brief, 4μm sections were mounted on silanized charged slides and allowed to dry for 10 min at room temperature and then for 20 min at65°C. After deparaffinization, heat-induced epitope retrieval using standard Cell Conditioning 1 was performed for 24 min. Subsequently, the primary anti-VSIG4 (1:50, cat. HPA003903, Sigma-Aldrich, St. Louis, USA) was labeled using an automated immunostaining system with the OptiView DAB Detection Kit (VentanaMedical Systems, Tucson, AZ, USA). Immunostained sections were counter-stained with hematoxylin.

**Figure 3:**
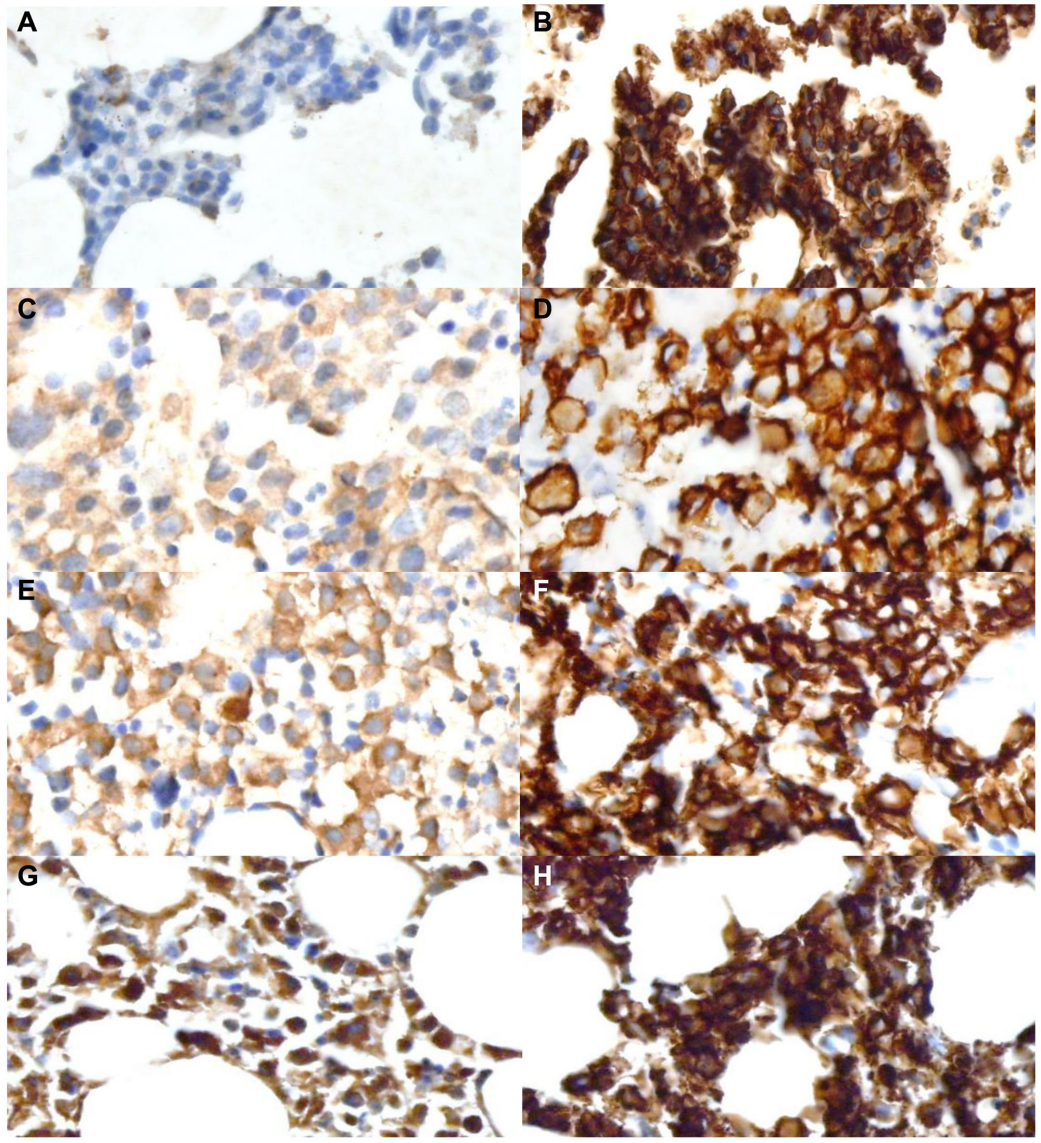
Immunohistochemical staining for the V-set Ig domain-containing 4 (VSIG4) and CD138 in representative tissue samples The intensity of VSIG4 immunostaining is arranged in increasing order. **(A)** Negative (0); **(C)** weak (1); **(E)** moderate (2); **(G)** strong (3). **(B, D, F, and H)** The tumor cells in the bone marrow biopsies were confirmed by immunohistochemical staining for CD138. Original magnification, ×400.

We examined the whole tissue section slides under a light microscope. The plasma cells were distinguished based on their characteristic appearance. CD138 IHC (1:100, MI-15, DAKO, Glostrup, Denmark) was also performed to confirm plasma cells. On VSIG4 IHC staining, the tumor cells showed membranous and cytoplasmic expression. Other cells in bone marrow except megakaryocytes did not show noticeable VSIG4 expression. Specific binding of VSIG4 antibody was validated on VSIG4 transfected human embryonic kidney 293T (HEK 293T) cell line (Supplementary Figure 1). The relative percentage of VSIG4-positive cells was counted and analyzed relative to the overall number of tumor cells. The labeling frequencies were quantified in 10% increments: 0, 0–10%; 1, 10–20%; 2,20–30%; 3, 30–40%; 4, 40–50%; 5, 50–60%; 6, 60–70%; 7, 70–80%; 8, 80–90%; and 9, >90%. The intensity of labeling was categorized as 0, 1, 2, or 3 for negative, weak, moderate, or strong staining, respectively (Figure 3). The total histologic score was calculated by multiplying the area score and the intensity score. For extramedullary biopsy, IHC was performed with the same protocol. Interpretation and quantification of the immunostained slides were also performed in the same manner as for bone marrow biopsies (Supplementary Figure 2). The histologic and IHC slides were reviewed independently by two pathologists who were blinded to the clinical information. In the case of discrepancy, a consensus was reached through discussion between the experts.

### Statistical analysis

The optimal cutoff value was determined using the Cutoff Finder which is an online accessible web application. Among variable methods for cutoff optimization, the hazard ratio plot was used for the analysis [39]. The clinical characteristics of the patients with low and high VSIG4 expression were compared by using the Chi-squared test (categorical variable) and Student's t-test (continuous variables). Survival curves were plotted using the Kaplan-Meier method, and the log-rank test was used to analyze the statistical differences between the life tables. The impact of VSIG4 expression on OS was analyzed by using univariate and multivariate Cox proportional hazard models. The proportional-hazards assumption was confirmed via examination of the log (-log [survival]) curves and no relevant violations were noted. *P*-values less than 0.05 were considered to indicate statistical significance. All statistical analyses were performed using the SPSS statistical software (version 21.0; IBM Corp., Armonk, NY, USA).

### Analysis of multiple myeloma genomics portal (MMGP) data

In order to corroborate the potential role of VSIG4 in MM, we analyzed a MMGP data set that contained survival information. The correlation between the expressions of protein and mRNA of VSIG4 is previously reported [40]. Genomic DNA and total RNA of primary tumors were obtained from CD138-enriched cell populations of bone marrow biopsy for the comparative genomic hybridization and mRNA microarray, respectively [41]. The data set included raw microarray gene expression data of 49 cases, based on results of Affymetrix microarrays. The expression of the *VSIG4* gene was analyzed and categorized as either high (log2(expression value) ≥ 8.3) or low (log2(expression value) <8.3). The OS and the EFS were calculated based on the information provided by the data set.

## SUPPLEMENTARY MATERIALS FIGURES AND TABLE


