# Supplementary material for: The immune checkpoint molecule V-set Ig domain-containing 4 is an independent prognostic factor for multiple myeloma
Source: Oncotarget. 2017 Jul 22;8(35):58122–32. doi: 10.18632/oncotarget.19468 (PMC5601638; doi:10.18632/oncotarget.19468)
Supplement: Supplementary file 1 [file oncotarget-08-58122-s001.pdf]

The immune checkpoint molecule V-set Ig domain-containing 4 is an independent prognostic factor for multiple myeloma

SUPPLEMENTARY MATERIALS

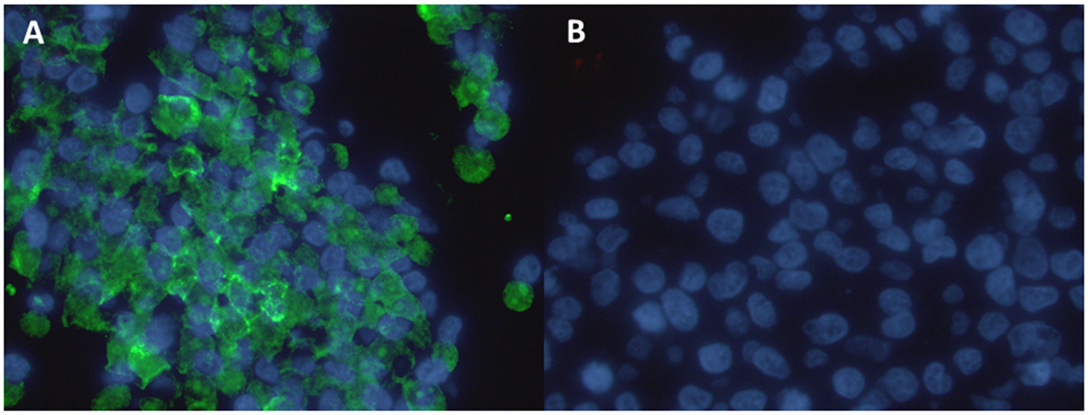

Supplementary Figure 1: Validation of specific binding for VSIG4 antibody. Specific binding of VSIG4 is validated on VSIG4 transfected human embryonic kidney 293T (HEK 293T) cell line **(A)**. We transfected HEK293 cells with plasmid (pcDNA3.1 (Invitrogen; Thermo Fisher Scientific, Inc.) encoding full-length of VSIG4 using Lipofectamin 2000 (Invitrogen; Thermo Fisher Scientific, Inc.). MOCK transfected HEK 293T cell line was used as negative control **(B)**. For immunofluorescent staining, anti-VSIG4 antibody (1:50, cat. HPA003903, Sigma-Aldrich, St. Louis, USA) was used for primary antibody and Fluorescein (FITC)-conjugated goat anti-rabbit IgG antibody (1:100, The Jackson Laboratory, ME, USA) was used for secondary antibody.

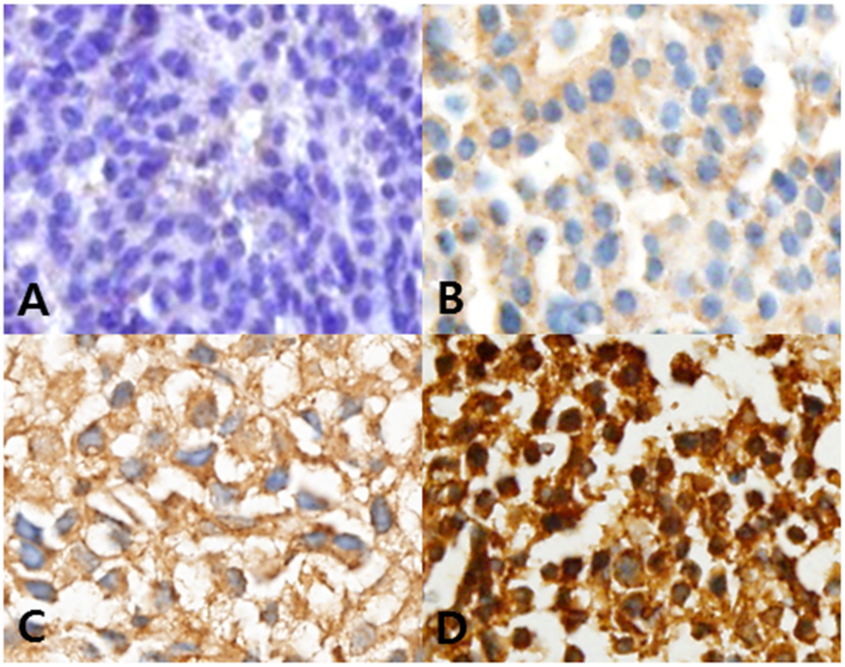

Supplementary Figure 2: Immunohistochemical staining of VSIG4 in extramedullary biopsies of multiple myeloma. The intensity of VSIG4 immunostaining is shown in increasing order. Higher magnification images showing cytomorphologic details are shown in the inset. **(A)** Negative (0); **(B)** weakly positive (1); **(C)** moderately positive (2); **(D)** strongly positive (3).

Supplementary Table 1: Characteristics of extramedullary myeloma patients and its correlation with VSIG4 expression.

		VSIG4		
	**Total (%)**	**Low (%)**	**High (%)**	**p value**
Age (years)				**0.01**
< 55	26 (39.4)	13 (19.7)	13 (19.7)	
≥ 55	40 (60.6)	8 (12.1)	32 (48.5)	
Sex				0.43
Male	33 (50.0)	9 (13.6)	24 (36.4)	
Female	33 (50.0)	12 (18.2)	21 (31.8)	
ISS				0.93
1	18 (27.3)	6 (9.2)	12 (18.5)	
2	36 (54.5)	12 (18.5)	24 (36.9)	
3	11 (16.7)	3 (4.6)	8 (12.3)	
Treatment				**< 0.01**
no ASCT	39 (59.1)	6 (9.1)	33 (50.0)	
ASCT	27 (40.9)	15 (22.7)	12 (18.2)	
Cytogenetics				0.39
Normal karyotype	36 (54.5)	12 (20.7)	24 (41.4)	
Abnormality	22 (33.3)	5 (8.6)	17 (29.3)	
Not done	8 (12.1)			
